# Characteristics of the Peritrophic Matrix of the Silkworm, *Bombyx mori* and Factors Influencing Its Formation

**DOI:** 10.3390/insects12060516

**Published:** 2021-06-02

**Authors:** Xu-Le Zha, Han Wang, Wei Sun, Hong-Yan Zhang, Jin Wen, Xian-Zhi Huang, Cheng Lu, Yi-Hong Shen

**Affiliations:** 1 State Key Laboratory of Silkworm Genome Biology, Southwest University, Beibei, Chongqing 400715, China; zhaxl1005@126.com (X.-L.Z.); wh18223@163.com (H.W.); hongyanzhang0321@163.com (H.-Y.Z.); 13032329491@163.com (J.W.); lucheng@swu.edu.cn (C.L.); 2 Laboratory of Evolutionary and Functional Genomics, School of Life Sciences, Chongqing University, Chongqing 401311, China; sunwei077@cqu.edu.cn; 3 Science and Technology Department, Southwest University, Chongqing 400715, China; hxz1166@swu.edu.cn

**Keywords:** *Bombyx mori*, peritrophic matrix, food, gut microbiota

## Abstract

**Simple Summary:**

The insect midgut is an important digestive organ with the peritrophic matrix (PM) being a semi-permeable membrane secreted by the midgut cells. The PM plays an important role in improving midgut digestion efficiency and protecting the midgut from food particles and exogenous pathogens. The silkworm, *Bombyx mori*, is an economically important insect. Understanding the structure of the PM is necessary for studying its function, but characteristics of PM in *B. mori* have been rarely reported. In this study, we conducted a comprehensive study on the PM structure of the PM in silkworms and found its thickness increased gradually during growth, but there was no difference in the thickness comparing the anterior, middle, and posterior regions. Permeability of the PM gradually decreased from the anterior to posterior regions. In addition, we found the formation of the PM was influenced by food ingestion and the gut microbiota.

**Abstract:**

The peritrophic matrix (PM) secreted by the midgut cells of insects is formed by the binding of PM proteins to chitin fibrils. The PM envelops the food bolus, serving as a barrier between the content of the midgut lumen and its epithelium, and plays a protective role for epithelial cells against mechanical damage, pathogens, toxins, and other harmful substances. However, few studies have investigated the characteristics and synthesis factors of the PM in the silkworm, *Bombyx mori*. Here, we examined the characteristics of the PM in the silkworms. The PM thickness of the silkworms increased gradually during growth, while there was no significant difference in thickness along the entire PM region. Permeability of the PM decreased gradually from the anterior to posterior PM. We also found that PM synthesis was affected by food ingestion and the gut microbiota. Our results are beneficial for future studies regarding the function of the PM in silkworms.

## 1. Introduction

In most insects, the midgut cells produce a semi-permeable membrane structure named peritrophic matrix (PM), which consists of chitin and proteins [1,2,3]. As a physical barrier, the PM protects the midgut from food, ingested toxins [4,5], and infection by pathogens [6,7,8,9,10]. It also separates the intestinal lumen into endoperitrophic and ectoperitrophic spaces for efficient nutrient acquisition and reuse of hydrolytic enzymes [11,12]. The biological role of the PM is highly related to its structural characteristics. For instance, after the PM structure in *Drosophila* is disrupted, toxin proteins released by bacteria are more likely to cross the PM and damage the midgut cells [4]. Pupal weight and adult emergence significantly are decreased in cases of PM structural alterations that impair digestion in *Spodoptera frugiperda* [13]. Deficiency of Insect Intestinal Mucin 3 in *Locusta migratoria* (LmIIM3) results in defects of the PM in the midgut and nymph lethality [14]. RNA interference (RNAi) of two peritrophic matrix proteins (PMPs) genes in *Tribolium castaneum*, *TcPMP3,* and *TcPMP5-B*, was shown to decrease PM thickness and increase PM permeability, eventually resulting in fat body depletion, growth arrest, molting defects, and mortality [15]. Several studies have suggested that antibodies specific to PM proteins impede insect development by binding their target antigens in the PM and blocking PM pores. In turn, nutrient uptake and/or the passage of digestive enzymes between the endo- and ectoperitrophic spaces were inhibited [16,17,18]. When the transcript levels of *chitinase* are reduced in the larval midgut of *Ostrinia nubilalis* by feeding-based RNAi, the chitin content in the PM is significantly increased; however, larval body weight is significantly decreased compared with that of control larvae fed a diet containing GFP dsRNA [19]. Together, these findings demonstrate the structural stability of the PM is essential for the growth and development of insects.

In general, there are two types of PM according to the mode of delamination [12,20,21]. Type I PM is derived from the midgut epithelium and found in most species of Coleoptera, Dictyoptera, Ephemeroptera, Hymenoptera, Odonata, Orthoptera, and Phasmida, as well as in lepidopteran larvae and blood-sucking dipteran adults [22,23]. Anti-peritrophin-55 antibody was used to immunolocalize the sites of PM synthesis in nine adult bee species. Teixeira et al. found that peritrophin-55 is produced in the rough endoplasmic reticulum of the midgut cells and released by secretory vesicles, which fuse with the plasma membrane and microvilli [24,25]. In *S. frugiperda*, peritrophin is released from microvilli of anterior midgut columnar cells [26,27,28]. In comparison, type II PM is produced by the cardia, a specialized tissue at the anterior region of the midgut found in some primitive insects, such as Dermaptera and isoptera, some lepidoptera, and the larvae of diptera [25,29].

The synthesis of PM is affected by many factors and can vary among different insects. For instance, the PM of many hematophagous insects occurs only after blood meal ingestion concomitant with the induction of genes encoding chitin synthase and PM-associated proteins or the release of pre-formed PM proteins from midgut epithelial cell [30,31]. In lepidopterans, the PM of starved larvae is thinner and more fragile compared to that of feeding larvae [32]. In post-molt larvae of Mamestra configurata that have just resumed feeding, the molting larval PM is replaced by a newer PM [33]. In contrast, the mRNA levels of the insect intestinal mucins (McIIM2–McIIM24) in M. configurata and the two peritrophins (peritrophin-57 and peritrophin-37) in Spodoptera litura are not altered after feeding or starvation [32,34].

The insect gut is a complicated ecosystem inhabited by a large number of microbes that play important roles in insect physiology and behavior [35,36,37]. It is now recognized as an “organ” that is indispensable for host health and the interactions between commensal bacteria and the vertebrate gut shown to modulate disease outcomes in multiple infectious, metabolic, and inflammatory disease models [38,39]. Elimination of gut microbiota in antibiotic-treated mosquitoes leads to PM loss and increased vectorial competence in *Anopheles stephensi*. Recolonization of antibiotic-treated mosquitoes with indigenous *Enterobacter sp.* restores PM integrity and decreases mosquito vectorial capacity [40]. In *Anopheles coluzzii* mosquitoes, the gut microbiota induces expression of several components of the PM, suggesting the microbiota are necessary for the synthesis of a structurally complete PM [41].

The silkworm, *Bombyx mori*, is a model insect of lepidoptera. However, there were few reports on the structural characteristics of the PM in silkworms and the factors influencing it. In the current study, we observed the surface structure of the PM at different developmental stages and measured its thickness and permeability. In addition, we found that PM synthesis was affected by food ingestion and the gut microbiota. Our results provide basic insight for illuminating the role of the PM in silkworms.

## 2. Materials and Methods

### 2.1. Insect Rearing

The silkworms (strain 305) were reared on fresh mulberry leaves and maintained at 25 ± 2 °C with 75% relative humidity under a photoperiod of 12 h light/12 h dark.

### 2.2. Thickness of PM

The midgut is equally divided into three segments, named anterior midgut, middle midgut, and posterior midgut. Corresponding to the midgut, the PM is divided into anterior, middle, and posterior sections. The anterior, middle, and posterior PM were extracted and were fixed in 4% paraformaldehyde overnight at 4 °C. Tissues were embedded in OCT (Cell Path, Newtown, UK) and sectioned at −20 °C using a cryotome (Thermo Scientific Inc., Waltham, MA, USA). Sections 5 µm thick were examined by optical microscope (Olympus, Tokyo, Japan). Ten measurements of 10 PM in silkworms were obtained using CellSens Dimension software.

### 2.3. PM Permeability

Larvae were fed mulberry leaves smeared with fluoresceine isothiocyanate (FITC)-dextran of different molecular masses (Sigma-Aldrich Corp., St. Louis, MO, USA). Midguts were dissected and divided into anterior, middle, and posterior regions; briefly, in order to prevent leakage of the FITC-dextran, we tied both ends of the anterior midgut together with surgical sutures and the anterior midgut was removed by forceps. Only the anterior PM was retained. The middle and posterior PM were obtained by the same method. The PMs were placed in embedding boxes containing OCT for 10 min, then frozen at −20 °C. The tissues were sectioned at −20 °C using a cryotome. Transverse 5 µm sections were analyzed using a fluorescence microscope (Olympus, Tokyo, Japan).

### 2.4. Transmission Electron Microscopy (TEM)

Midguts were dissected from the silkworms and equally divided into anterior, middle, and posterior sections. The tissues were fixed in 4% paraformaldehyde overnight at 4 °C. The samples were then dehydrated in a graded acetone series before infiltration and embedding in Eponate 12 resin (Ted Pella, Redding, CA, USA). The resin-embedded specimens were baked at 60 °C for 48 h. All specimens were sectioned into 60 nm thick sections using an ultratome. The samples were stained in dark with uranyl acetate for 10 min and then stained with lead citrate for 10 min. Samples were examined using a Hitachi HT7700 TEM (Hitachi, Tokyo, Japan).

### 2.5. Scanning Electron Microscopy (SEM)

The PM was removed from the midgut and placed on a coverslip. The PM was opened with scissors, rinsed with phosphate-buffered saline (PBS) to remove food particles, and then dehydrated in a graded ethanol series before being dried in a graded tert-butanol and acetonitrile series. Samples were sputter-coated with gold and examined using a Hitachi SU3500 SEM (Hitachi, Tokyo, Japan).

### 2.6. Antibiotic Treatment

Fresh mulberry leaves were cut into 1 cm × 1 cm pieces. A 10 μL dose of chloramphenicol dissolved in absolute ethanol (30 mg/mL) was applied onto each piece of leaf for the treatment group. An equal volume of absolute ethanol was added to the surface of mulberry leaves as a control. One piece of antibiotic-treated or mock-treated leaf was supplied to each larva until the larva thoroughly consumed the piece of leaf, and then provided the silkworms with fresh mulberry leaves.

### 2.7. Quantitative Reverse Transcription Polymerase Chain Reaction (qRT-PCR)

To monitor mRNA levels of genes in silkworms, qRT-PCR experiments were performed. Bacterial load was evaluated by qRT-PCR amplification of the 16S rRNA gene. Total RNA was isolated from each sample by using TRIzol reagent (Thermo Fisher Scientific Inc., Waltham, MA, USA) and 2 µg of total RNA was reverse transcribed into complementary DNA (cDNA) using a reverse transcription kit (Takara, Shiga, Japan). The qRT-PCR amplifications were performed in duplicate using SYBR™ Select Master Mix (Bio-Rad, Hercules, CA, USA) in a total volume of 10 μL on a qTOWER3G Real-Time PCR system (Analytikjena, Berlin, Germany). Eukaryotic translation initiation factor 4A (microarray probe ID: sw22934) was used as an internal control [42]. Samples were collected with three biological replicates, each with five silkworms. The experiment was repeated thrice with the biological and technical replicates. All the primers used are listed in Table 1.

### 2.8. Statistical Analysis

All data were statistically analyzed using t-tests. Data from at least three independent experiments are presented as means ± SEM. Statistical significance was set at *p* < 0.05.

## 3. Results

### 3.1. Measurement of PM Thickness at Different Development Stages of Silkworm

The thickness of the anterior, middle, and posterior PM were measured on the 2nd day of the third instar, 2nd day of the fourth instar, and 3rd day of the fifth instar. The results showed that the PM thickness on the 2nd day of the third instar was 9.13 ± 1.55 μm in the anterior region, 9.42 ± 1.35 μm in the middle region, and 9.85 ± 0.06 μm in the posterior region (Table 2). On the 2nd day of the fourth instar, the PM thickness was 9.33 ± 0.85 μm in the anterior region, 9.67 ± 0.54 μm in the middle region, and 9.89 ± 1.23 μm in the posterior region (Table 2). The PM thickness of the anterior, middle, posterior regions on the 3rd day of the fifth instar were 12.59 ± 1.08 μm, 11.86 ± 1.35 μm, and 11.52 ± 0.79 μm, respectively (Table 2). The PM of the fifth instar larvae was significantly thicker than that of the third and fourth instar larvae. These results showed the thickness of the PM gradually increased with growth. However, there were no significant differences in PM thickness among the anterior, middle, and posterior regions of different development stages of the silkworm.

### 3.2. PM Permeability and Microvilli Morphology of Different Midgut Regions in Silkworm

**Figure 1:**
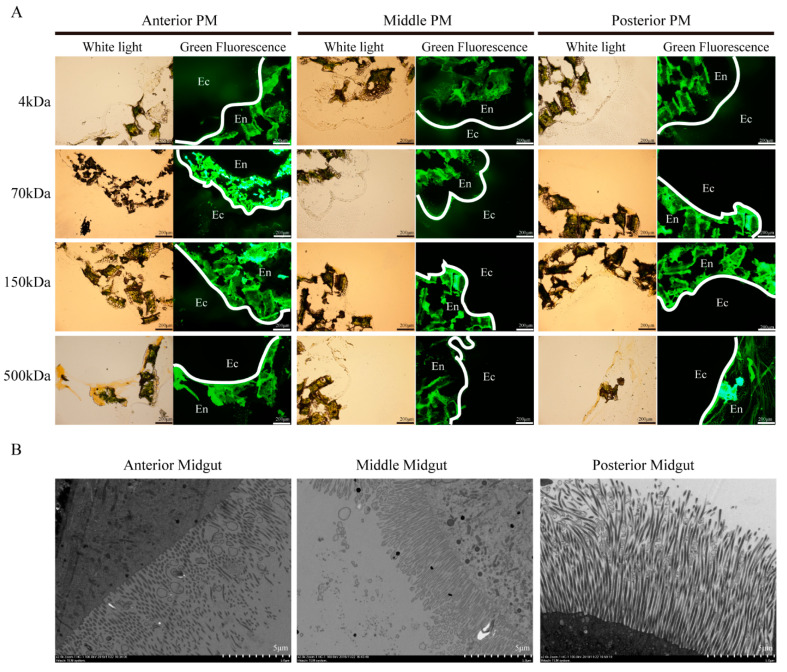
Peritrophic matrix (PM) permeability and microvilli morphology of silkworms in different regions of the larval midgut. (**A**) Anterior, middle, and posterior PM permeability on the 3rd day of the fifth instar larvae detected using fluoresceine isothiocyanate (FITC)-dextran and fluorescence microscope (100×). En, endoperitrophic space. Ec, ectoperitrophic space. White lines indicate the location of the PM. (**B**) Microvilli and secreted vesicles of the anterior, middle, and posterior midgut on the 3rd day of the fifth instar larvae evaluated using transmission electron microscopy (2000×).

FITC-dextran with different molecular sizes were used to evaluate PM permeability in the silkworm on the 3rd day of the fifth instar. The results showed that FITC-dextran with a molecular weight of 4 kDa was detected in the ectoperitrophic space, whereas the 500 kDa FITC-dextran was restricted to the endoperitrophic space (Figure 1A). The 70 kDa FITC-dextran was able to pass through the anterior and middle PM, but could not pass through the posterior PM (Figure 1A). The 150 kDa FITC-dextran could cross the anterior PM, but it could not cross the middle and posterior PM (Figure 1A). Therefore, we concluded that the permeability of the PM in silkworm on the 3rd day of the fifth instar decreased from the anterior through to the posterior PM regions with the anterior PM permeability cutoff being <500 kDa, the middle PM being <150 kDa, and the posterior PM being <70 kDa.

The ultrastructure of the silkworm midgut on the 3rd day of the fifth instar was also evaluated using TEM. There were significantly fewer microvilli and secreted vesicles in the anterior and middle midgut compared to those in the posterior midgut (Figure 1B). We speculated that the proteins in vesicles merged into the PM and participated in the synthesis of the PM, which may be one reason the permeability of the posterior PM was lower than that of the anterior and middle PM.

### 3.3. Silkworm PM Surface Structure at Different Developmental Stages Evaluated by SEM

**Figure 2:**
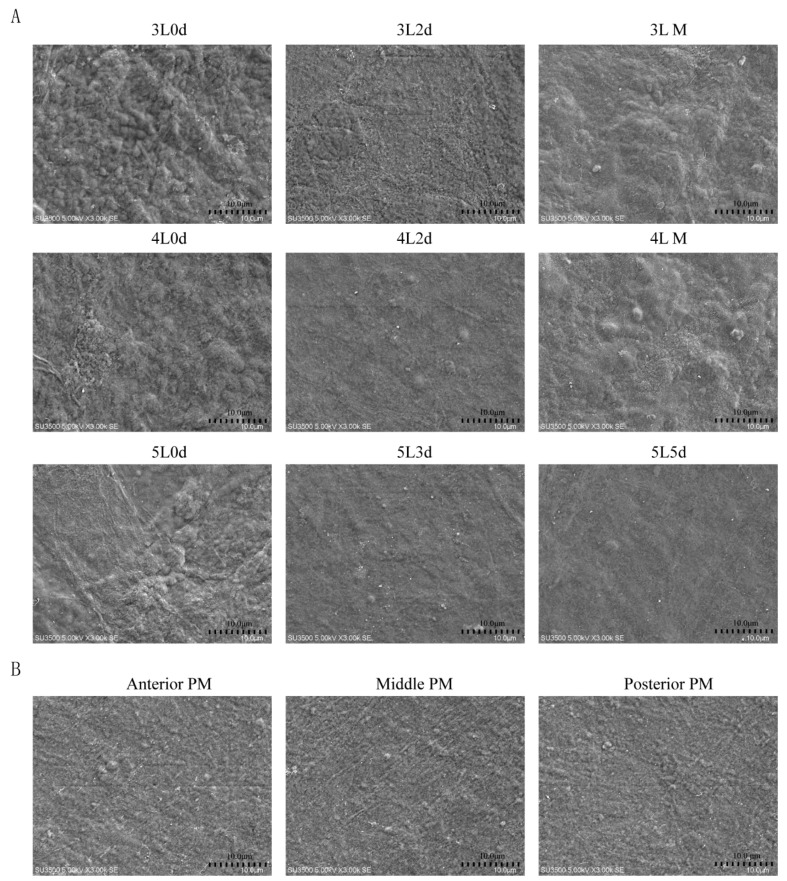
Morphological characterization of the lumen side of the peritrophic matrix (PM) in silkworm was observed by scanning electron microscopy. (**A**) Morphological variation of the middle PM in different development stages of silkworm (3000×). nL, instar of larval stage “n”; M, Molt; nd, Day ”n”. (**B**) Morphological characterization of anterior, middle, and posterior PM on the 3rd day of the fifth instar larvae (3000×).

Our previous study showed that *peritrophins* are more abundant during feeding than during molting. The mRNA expression levels of *peritrophins* gradually increase with development [43]. The surface structure of the middle PM of silkworms was observed at different developmental stages (day 0 of the third instar through to the 5th day of the fifth instar) using SEM. The results showed that the surface of the PM was rough and loose during molting and new molted stages, but smooth and dense during the feeding period (Figure 2A). However, no significant differences were observed among the anterior, middle, and posterior PM surface structures on the 3rd day of the fifth instar (Figure 2B).

### 3.4. Silkworm PM Synthesis May Be Induced by Food Ingestion

**Figure 3:**
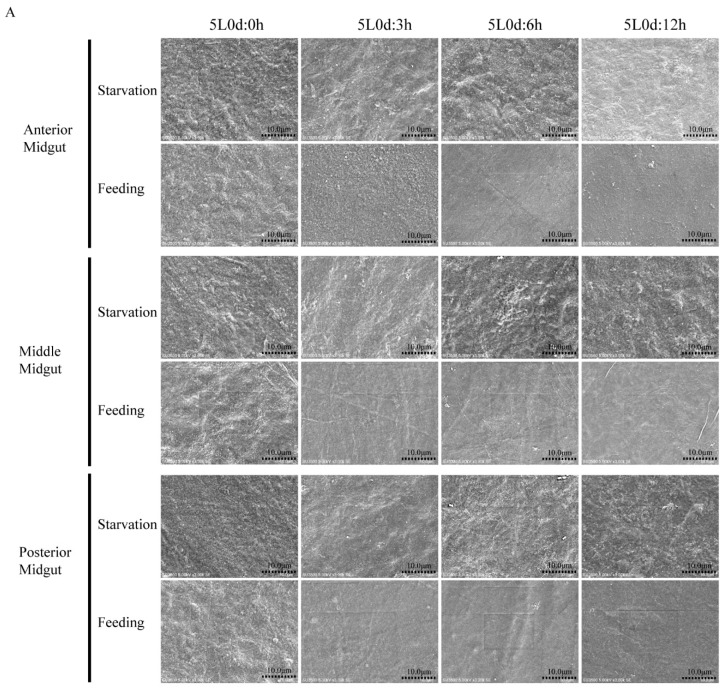

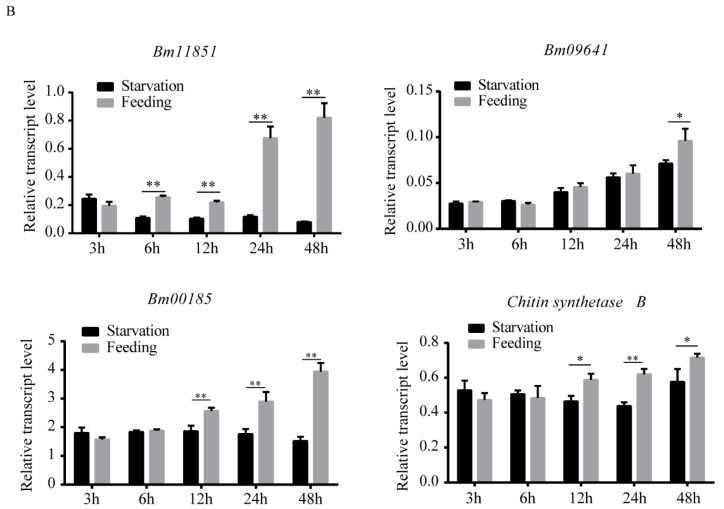
Restoration of the peritrophic matrix (PM) structure may be promoted by food ingestion. (**A**) Morphological changes of the lumen side of the PM in silkworm larvae were observed at different times during starvation and feeding using scanning electron microscopy (3000×). 5L0d, day 0 of the fifth instar. (**B**) Expression of four genes encoding components of the PM in silkworm on day 0 of the fifth instar larvae at different times post-initiation of starvation and feeding. * *p* < 0.05, ** *p* < 0.01.

A previous study found that Ag-Aper1 and Ag-Aper14 in *Anopheles gambiae* accumulated in the PM during blood sucking and was involved in PM synthesis [30,31]. To investigate the influence of food ingestion on PM formation, the surface structure of the PM was observed by SEM on day 0 of the fifth instar larvae at different times during starvation and feeding. The surface structure of the PM on day 0 of the fifth instar was loose and rough and it remained rough following prolonged starvation (Figure 3A). When the silkworms were fed mulberry leaves for 3 h, the anterior, middle, and posterior PM structures became dense and smooth, speculating a restoration of the PM structure was induced by food ingestion (Figure 3A). We again analyzed the mRNA expression levels of three *peritrophins* and one *chitin synthetase* in silkworms on day 0 of the fifth instar larvae at different times during starvation and feeding. There were no differences in the expression of *peritrophins* and *chitin synthetase* at 3 h post-initiation of feeding compared with that after 3 h of starvation (Figure 3B). We hypothesized these PM proteins were already stored in the midgut cells, and that these genes were preferentially induced for PM synthesis when the silkworms were fed mulberry leaves. The expression of *peritrophins* and *chitin synthetase* in the midgut gradually increased with the prolongation of feeding time, indicating the midgut cells may synthesize PM proteins for use in PM formation (Figure 3B).

### 3.5. Gut Microbiota Play a Role in Maintaining PM Structural Integrity in Silkworm

**Figure 4:**
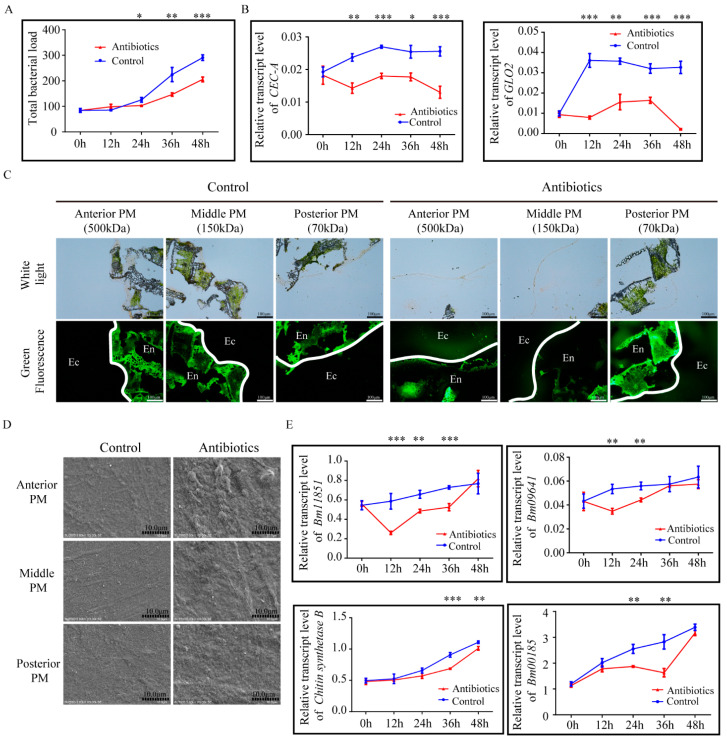
Antibiotic treatment compromises the integrity of the peritrophic matrix (PM) in the silkworm. (**A**) Bacterial load in the guts of control and antibiotic-treated silkworms detected by qRT-PCR using universal 16S ribosomal RNA (rRNA) primers. * *p* < 0.05, ** *p* < 0.01, *** *p* < 0.001. (**B**) Relative expression levels of antimicrobial peptide genes *CEC-A* and *GLO2* in the midgut of control and antibiotic-treated silkworms. (**C**) Permeability of the anterior, middle, and posterior PM 24 h post-feeding with or without antibiotic supplementation evaluated by fluorescence microscope (200×). En, endoperitrophic space; Ec, ectoperitrophic space. White lines show the localization of the PM. (**D**) Morphological changes in the lumen side of the PM of control and antibiotic-treated silkworms was observed by scanning electron microscopy 24 h post-feeding (3000×). (**E**) Expression of four genes encoding components of the PM in the midguts of control and antibiotic-treated silkworms. ** *p* < 0.01, *** *p* < 0.001.

It has been previously reported that gut microbes promote PM structural integrity [40,41]. Therefore, we analyzed whether gut microbiota impacted PM structure in the silkworm. Chloramphenicol (0.3 mg) was fed to 1st day of 5th instar larvae. The antibiotic treatment was effective at substantially depleting gut bacteria in the silkworms by 24 h post-treatment, according to the qRT-PCR analysis of bacterial 16S rRNA. (Figure 4A). Correspondingly, the expression of antimicrobial peptide genes (*CEC-A* and *GLO2*) was also significantly reduced (Figure 4B). FITC-dextrans with different molecular weights were used to evaluate PM permeability in the silkworm 24 h after antibiotic treatment. Permeability of the anterior, middle, and posterior PM increased significantly (Figure 4C).

In addition, PM surface structure in the silkworms was also evaluated after 24 h of antibiotic treatment using SEM. The anterior, middle, and posterior PM became rough and loose compared to that in the control group (Figure 4D). Analysis of the expression of the four PM genes revealed *Bm11851* expression was significantly reduced from 12–36 h after antibiotic treatment, *Bm09641* expression was significantly reduced from 12–24 h after antibiotic treatment, *chitin synthase B* expression was significantly reduced from 36–48 h after antibiotic treatment, and *Bm00185* expression was significantly reduced from 24–36 h after antibiotic treatment (Figure 4E). Thus, gut microbes may play a role in regulating the expression of PM-associated genes, thereby helping to maintain silkworm PM structural integrity.

## 4. Discussion

The PM is a semi-permeable membrane secreted by the midgut epithelium of most insects and includes type I and type II PM. Silkworm is a model insect of lepidoptera with larvae containing type I PM. As the first physical barrier, the PM plays an important role in protecting the midgut from food particles, resisting an invasion of exogenous bacteria and harmful substances, improving midgut digestion efficiency, and maintaining intestinal homeostasis [11,40,41,44,45,46,47]. To study the function of the PM in silkworms, we systematically studied the biological characteristics of the PM in silkworms.

We found that PM thickness in silkworms gradually increased with growth, but there were no significant differences in PM thickness among the anterior, middle, and posterior PM of each instar. It was previously reported that PM thickness gradually increases from the anterior to posterior PM in *O. nubilalis* [48], *Manduca sexta* [49], *Anomala cuprea* [24], *T. castaneum* [15], *Melipona quadrifasciata*, and *Apis mellifera* [18]. The thicker PM in the posterior midgut regions of these species may be due to PM accumulation in the midgut region, which has been reported for *T. castaneum* (Coleoptera) [15]. However, it was difficult to explain why there were no differences in the thickness of the anterior, middle, posterior PM of the silkworms.

Permeability is an important feature of the PM and is essential in terms of digestive processes, including digestive compartmentalization [18,50]. Our current results showed the posterior PM of the silkworm exhibited lower permeability compared to that of the anterior and middle PM regions. Similar results have been reported for *T. castaneum* where PM permeability progressively decreased along the midgut from the anterior to the posterior region [15]. In contrast, the PM of *Rhynchosciara americana* [11], *A. cuprea* [24], *M. quadrifasciata*, and *A. mellifera* [18] had similar permeability throughout their midguts. Similar PM permeability with different PM thickness may be due to specific maturation processes in the various midgut regions [18].

We also found that the numbers of microvilli and secreted vesicles in the posterior midgut of silkworms were significantly higher compared to those in the anterior and middle midgut based on TEM. Levy et al. previously reported *Anticarsia gemmatalis* larvae that are resistant to *Anticarsia gemmatalis* multiple nucleopolyhedrovirus secreted more vesicles in the posterior midgut than that in the anterior and middle midgut [51]. Peritrophins have also been shown to be released from double-membrane vesicles budding from the microvilli of the anterior midgut of *S. frugiperda* [27,52]. We speculate there were a large number of PM proteins present in the vesicles secreted in the posterior midgut and the PM proteins involved in the synthesis of the PM, which may be one of the reasons for the low permeability of the posterior regions.

The PM structure was dense and smooth during the feeding stage, but loose and coarse during the molting stage and new molted stage. Previous studies have found that the expression of *peritrophins* is higher during the feeding period compared to that during the molting period and just after molting [43]. The PM surface structure in silkworm on day 0 of the fifth instar was loose and rough when starvation conditions were initiated, and the expression of *peritrophins* and *chitin synthetase* in the midgut remained unchanged. However, the PM structure became smooth and dense after 3 h of feeding and the expression of *peritrophins* and *chitin synthetase* in PM gradually increased for 6 h after feeding, indicating PM synthesis was affected by the ingestion of food. However, compared with that of the starvation group, the expression of genes responsible for PM synthesis did not significantly change for 3 h after feeding. We speculate that proteins related to PM synthesis were stored in the midgut cells and would then preferentially participate in the formation of PM when stimulated by the ingestion of food. It has been reported that Ag-Aper14 is asymmetrically localized toward the luminal side of epithelial cells in the posterior midgut of *A. gambiae* after sugar feeding, but is secreted into the ectoperitrophic spaces after 24 h blood sucking in order to participate in the formation of PM [30,31]. Interestingly, neither feeding nor starvation has an effect on mRNA levels of *peritrophins* in *S. litura* or insect intestinal mucins (IIMs) in *M. configurata* [32,34].

Microbiota in the gut of insects has a wide range of effects on host nutrition, physiology, metabolism, and degradation of harmful substances, host growth and development, immune response, and defense against pathogens [37]. Recent studies have suggested that gut microbiota may affect PM synthesis. The PM structure in the silkworms became rough and loose after treatment with chloramphenicol and its permeability increased, indicating the PM was structurally damaged. Moreover, the expression of *peritrophins* and *chitin synthetase* in the midgut was significantly reduced following antibiotic treatment. When the gut microbiota of *A. coluzzii* and *A. stephensi* is removed by antibiotic treatment, their PM structures are disrupted, but obvious PM structures are restored in mosquitoes supplemented with *Enterobacter* sp. [40,41]. Meanwhile, *Ixodes scapularis* larvae fed the blood of gentamicin-treated mice have a significantly thinner PM in their midgut compared to that in larvae fed the blood of untreated mice [38]. These results suggest that the presence of the microbiota is required for the synthesis of a structurally integrated PM. However, the mechanism between PM and gut microbiota has not been reported. The gut microbiota is very complex, including commensal, pathogenic, and parasitic microbes, and so on. We hypothesized that when the number of harmful bacteria increases, the PM will become thicker or denser to resist bacterial invasion. In contrast, when antibiotics remove the gut microbiota, the PM does not need to prevent the invasion of harmful bacteria, so the PM structure becomes looser.

## 5. Conclusions

**Figure 5:**
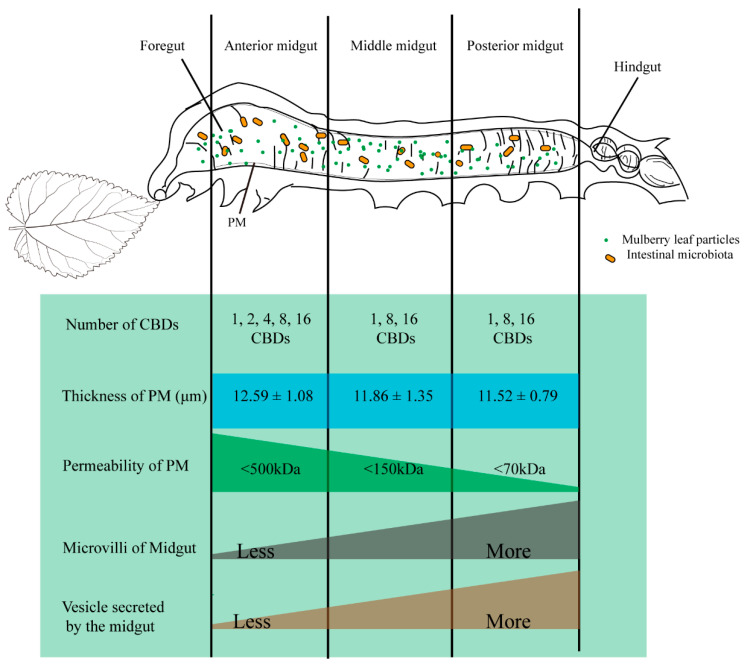
Model of the peritrophic matrix (PM) structure on the 3rd day of the fifth instar in silkworm, *Bombyx mori*. Our previous study found that peritrophins with different amounts of chitin-binding domain varied in their distribution in the silkworm midgut [43]. Significant thickness differences among the anterior, middle, posterior midgut were not observed. PM permeability progressively decreased along the midgut from the anterior to posterior region. Finally, there were more microvilli and secreting vesicles in the posterior midgut of the silkworms compared to those in the anterior and middle regions of the midgut.

We comprehensively studied the characteristics of the PM of silkworms and obtained a model of the PM structure (Figure 5); the PM thickness of silkworms increased gradually with growth, while there was no significant difference in the thickness of the whole PM region. The microvilli and secretory vesicles in the midgut gradually increased from the anterior to the posterior region. This may be one of the reasons why PM permeability decreases gradually from the anterior to posterior PM. In addition, PM synthesis is affected by the food and gut microbiota.

## Figures and Tables

**Table 1:** Primer sequences for qRT-PCR in this study.

Name	Forward (5′→3′)	Reverse (5′→3′)
*Bm09641*	CTGAAGGTTCGGGCTTGGGT	TGTGCCTGCTGAGTCTGCTGTG
*Bm01504*	TGGCCTCAGAATGTCGACT	CAATAATCTAAAATCCATAATGCTAC
*Bm00185*	CATCCTCCCCTGGGCTCAC	CGTAATCAAGGTCATTTGTTCGC
*Bm11851*	GCAGAACAGGTTTGCGACTG	GCTCAGGCTCTTGTTCTGGT
*Bm01491*	AAAGCTCCAGGGAGACAACG	TCCTCACCTGGAACGACTCT
*sw22934*	TTCGTACTGGCTCTTCTCGT	CAAAGTTGATAGCAATTCCCT
*GLO2*	CTAAATAGACAAATCGGTGGC	GCGGATCTCTGCTTGAAGAC
*CecA*	CTTCGTCTTCGCGTTGGT	AAGGATTTCGCTTGCCCTAT
*16sRNA*	TACGGGAGGCAGCAG	ATTACCGCGGCTGCTGG

**Table 2:** Peritrophic matrix (PM) thickness at different developmental stages of the silkworm, *Bombyx mori*.

		PM Thickness [μm] (Mean ± SEM)		
Instar	Number of PMs	Anterior PM	Middle PM	Posterior PM
Third instar	20	9.13 ± 1.55 ^b^	9.42 ± 1.35 ^b^	9.85 ± 0.06 ^b^
Fourth instar	20	9.33 ± 0.85 ^b^	9.67 ± 0.54 ^b^	9.89 ± 1.23 ^b^
Fifth instar	20	12.59 ± 1.08 ^a^	11.86 ± 1.35 ^a^	11.52 ± 0.79 ^a^

Different letters represent significant differences.

## Data Availability

Data is contained within the article.
